# Lessons learned using species’ distribution models for conservation planning in the Golden Gate Biosphere reserve

**DOI:** 10.1371/journal.pone.0343037

**Published:** 2026-03-11

**Authors:** Alexandra D. Syphard, Heather Rustigian-Romsos, Daniel Franco, Alison Forrestel

**Affiliations:** 1 Conservation Biology Institute, Corvallis, Oregon, United States of America; 2 Golden Gate National Parks Conservancy, San Francisco, California, United States of America; 3 Golden Gate National Recreation Area, San Francisco, California, United States of America; HUN-REN Centre for Ecological Research, HUNGARY

## Abstract

Conservation practitioners responsible for maintaining biodiversity and ecosystem services within protected areas require information about how dominant plant species may reassemble under rapid global change. Although species’ distribution models (SDMs) alone do not account for multiple threats or species population dynamics, they can provide robust assessments of where species may persist or disperse to in the future, especially if carefully constructed and thoroughly evaluated. We used ensemble SDMs to evaluate how climate change may alter suitable habitat for six dominant plant species representing different life forms within the Golden Gate Biosphere Network (GGBN), located along the central to northern coast of California. We trained the models on presence-absence data and 23 environmental predictors, including climate, topography, and soils, using six algorithms. We projected habitat suitability to late-century climate conditions using three GCMs under RCP 8.5 and summarized areas of agreement, expansion, and refugia. Model results can be summarized into several lessons learned, many of which are consistent with previous research. The first is that projected habitat may either expand or contract, and the direction of change varies by individual species, even within the same genus. Many projected changes reflected species’ relationships with key climate variables relative to their future projected trends. Disagreement across scenarios was largely driven by uncertainty in projected precipitation changes, while non-climatic variables, particularly soils, were also important in mediating projected habitat change. Contrary to common assumptions, projected habitat shifts were not always upslope or poleward. Finally, although species face multiple threats from other global changes, SDMs can provide a valuable baseline for conservation decisions within small reserves, particularly through identification of refugia and comparison of model scenarios. However, habitat changes within protected areas may not reflect dynamics elsewhere across species’ ranges, underscoring the need for multi-scale conservation planning.

## Introduction

The potential for plant species’ range shifts in response to climate change is one of the most pressing biodiversity conservation concerns [1]. Not only may rapidly changing temperature and precipitation regimes directly exceed species’ physiological limitations, but climatic effects on species’ interactions and altered community structure may also interfere with establishment and survival [2]. Furthermore, species may face interacting or synergistic effects from other global processes, such as land use change and altered disturbance regimes [3]. These risks are especially pronounced if disturbances occur in areas where climatic conditions are no longer suitable for the plant species existing on the site (e.g., vegetation-climate mismatch) [4]. Given these complexities, projecting species’ range shifts under climate change is a critical challenge in conservation planning. Amidst the complexities of projecting future conditions, conservation planners are tasked with anticipating how and where to focus management efforts, often within the specific boundaries of protected areas.

The most prevalent means for projecting climate change impacts to individual species’ ranges, and collectively to biodiversity, has been species distribution models (SDMs) [5]. SDMs employ statistical correlational approaches to estimate species’ response functions to climatic and other environmental variables that influence habitat suitability [6]. Derived from observed species’ locality and mapped environmental data, fitted models conditioned upon current environmental conditions are used to map habitat suitability at unsurveyed locations [7], and to project potential range shifts by substituting future climate projections with current climate variables used to fit the SDM [8]. These habitat suitability projections are often considered a first approximation of species’ exposure to and risk from climate change [1].

The increased use of SDMs for range-change applications and research is due in part to their relatively low cost and ease of use, for example using free software packages (e.g., [9]). Data availability both in terms of species’ occurrence locations and mapped predictor variables has also been growing rapidly, often open access [10]; and model output is relatively easy to interpret [6]. Despite these advantages, SDMs have also been criticized for potential data or model uncertainty, high sensitivity to model parameterization, insufficient accounting for dispersal or population dynamics, and inability to incorporate feedbacks with other threats, such as land use or disturbance, or biotic interactions [11]. In short, there are calls to move beyond SDMs for predicting biodiversity responses to global change [1]. In response to these concerns, some newer and more sophisticated modeling approaches have been developed. For example, some approaches integrate SDMs with additional model types to account for combined effects of habitat suitability change, species’ demographics, land use change, and altered disturbance regimes (e.g., [12,13]).

While these more sophisticated approaches introduce more complexity and potential realism into projections, many conservation organizations lack the budget or qualified expertise to implement these methods. Nevertheless, there has also been substantial research investment into improving the robustness and accuracy of SDMs. For example, numerous studies documenting the largest sources of uncertainty in the modeling process (e.g., modeling method, predictor variables, climate models, spatial and temporal scale, species’ traits, and biogeographical characteristics) have consistently shown that the SDM modeling algorithm has the largest influence on model performance (e.g., [6,14,15]). Individual model types result in large variation in both spatial predictions (e.g., [16–18]) and model performance [19].

In response to the uncertainty produced by using different model types, consensus methods have been developed, which combine results from several model types into an ensemble of predictions. Ensemble modeling involves the construction of multiple SDMs using different modeling algorithms and synthesizing results based on one of several approaches [20–22]. In addition to creating ensembles, careful selection of predictor variables, ensuring statistical model assumptions are met, and documenting data sources and other sources of uncertainty can help to ensure that SDMs are used appropriately relative to the objectives of the application [23]. Carefully constructed SDMs, therefore, remain important tools for conservation planning and for addressing research questions regarding climatic impacts on habitat change and biodiversity.

One of the biggest challenges in understanding how biodiversity may be impacted by global change is that responses to climate and other global changes are likely to vary depending on individual species. Thus, despite concerns over habitat loss under climate change, prior studies (e.g., [24]) and past evidence (e.g., [25]) suggest that future habitat could also expand depending upon species’ climatic tolerances and locations relative to the pace and magnitude to climate change exposure [26,27]. SDMs could therefore serve as important tools for estimating how the habitats of multiple species may reassemble in the future relative to existing protected areas [28].

Of particular concern is how vegetation communities may shift in the future. While the distribution of rare and endemic species is a great concern for biodiversity conservation, dramatic change in the distribution of dominant life forms, or vegetation types, can result in dramatic or cascading shifts in ecosystem structure and function [4,29,30], ultimately affecting humans through provisioning of ecosystems services [31]. Because species respond individualistically to climate change, existing vegetation communities or assemblages may not persist as is into the future. Thus, modeling dominant species that represent vegetation communities or habitat types offers a potential surrogate for estimating the broader-scale impacts of range changes [32,33].

A further unresolved question is the extent to which species’ range changes shift in predictable directions. Given the lower temperatures associated with higher elevations and latitudes, a common assumption is that species’ habitat suitability and thus ranges will shift in those directions with a warming climate [34–36]. However, some research suggests that, particularly in complex terrain with fine-scale microclimates, that refugia may be found in other locations [37–39]. In California, climate change exposure is also strongly mediated by the distance to the coast [40]. Knowing how the habitats of dominant species may change relative to elevation, topographic position, or latitude may help to anticipate whether certain species’ characteristics or life forms may influence their persistence via refugia on the landscape.

**Fig 1:**
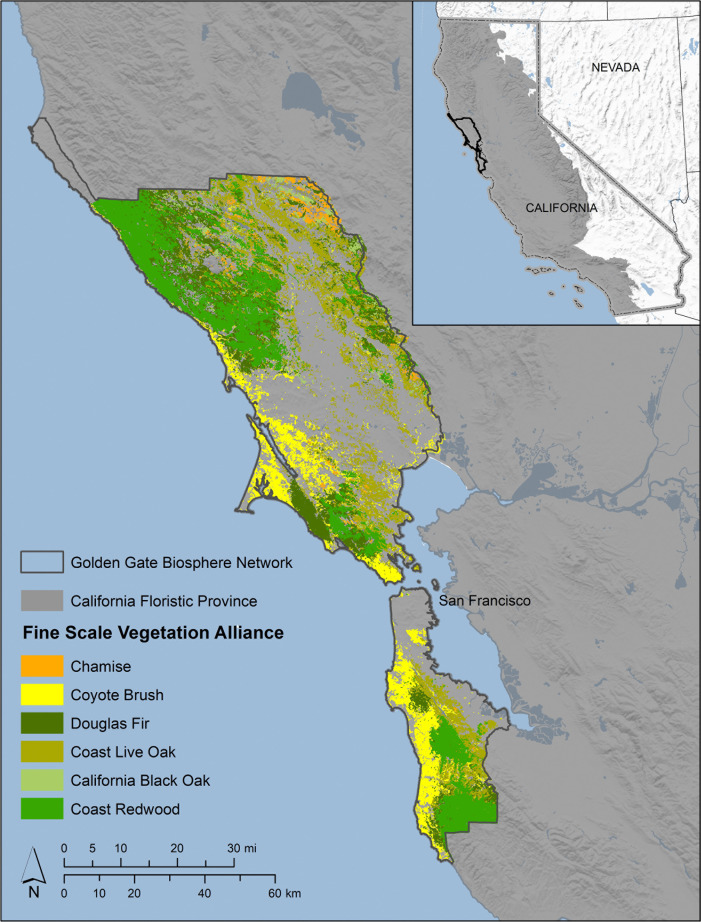
Vegetation alliances within the Golden Gate Biosphere Network. The inset map depicts the location of the California Floristic Province (dark gray) and the Golden Gate Biosphere Network terrestrial boundary (black outline) in California.

While SDMs are often used at broad scales across entire species’ ranges, often for prioritizing areas to establish conservation reserves [41–43], other conservation practitioners are tasked with managing lands within established boundaries, sometimes within small reserves without much area to accommodate adaptation. One regional-scale reserve with high conservation value is the Golden Gate Biosphere Network (GGBN), located along the central to northern coast of California (Fig 1). This area supports high biodiversity within a complex mosaic of habitat types, with many species being rare or endemic to the state. Tasked with protecting the sensitive natural resources in this region, it is important for the Golden Gate Biosphere Network to assess and anticipate the potential for large-scale habitat shifts in response to climate change. This information is critical for conservation management decision making, including prioritizing areas for restoration that are likely to function as climate refugia, identifying areas where dominant vegetation types are likely to shift, and recognizing where resistance-based management may be ineffective. The approach presented in this study is intended as a practical model for local and regional scale conservation managers seeking to prioritize conservation actions under climate change.

We illustrate a methodological approach for using SDMs and ancillary data to inform conservation planning and to address several research questions about the nature of range changes. Specifically, we used ensemble SDMs to evaluate how climate change may alter the distribution and occurrence of six priority plant species within the GGBN. Our objectives were to identify and prioritize locations of potential refugia for conservation and management action and to answer these research questions:

1) Will projected climate change reduce suitable habitat for all species, and if not, which life forms are expected to persist or expand?
2) Will projected habitat expansion or contraction vary according to climate change scenarios?
3) What are the mean characteristics of projected climate refugia, and how do they vary for different species? Will habitat shift upward in elevation or higher in latitude as often expected?

## Methods

### Study area

The Golden Gate Biosphere Network, was designated by the United Nations Educational, Scientific, and Cultural Organization (UNESCO) Man and the Biosphere Program (MAB) in 1988 (https://www.goldengatebiosphere.org/) to protect the San Francisco Bay Area’s ecological and cultural resources. A diverse partnership of governmental agencies (local, state, federal, and tribal), nonprofits, universities, community organizations, and the private sector collaborate to conserve the region’s biodiversity and promote sustainable development. We focus here on the terrestrial portion of the network, which includes protected, multi-use managed, and urban areas. The terrestrial portion of the GGBN spans more than 6900 km^2^, with elevations ranging from sea level to 1327 m. Important plant communities within the GGBN include coast redwood forests, mixed evergreen forests, oak woodlands, coastal scrub, and chaparral.

### Priority plant species

The project manager and collaborators working in the GGBN selected six priority plant species representing the broad range of community types and lifeforms present in the region. These species were selected because each one is a dominant species within one of the major plant communities within the project area. These included two shrubs (coyote brush (*Baccharis pilularis*) and chamise (*Adenostoma fasciculatum*). Coyote brush is the dominant species of the coastal scrub plant community which covers 87039 acres (352 km^2^) within the project area, of which 56% is protected (S1 Table). Chamise is a dominant chaparral species which, covers 15500 acres (63 km^2^) within the project area, of which 23% is protected, and is projected to expand in range by other climate modeling efforts for the region [33]. We also selected two conifer trees (coast redwood (*Sequoia sempervirens*) and Douglas fir (*Pseudotsuga menziesii*)). Coast redwood forest covers 162675 acres (658 km^2^) within the project area, 30% of which is within protected lands, and is an iconic species for visitor experience and conservation. Douglas fir is a dominant species in coastal mixed evergreen forests which cover 158450 acres (641 km^2^) of the project area, 34% protected. Finally, we chose two oak species, coast live oak (*Quercus agrifolia*) and California black oak (*Quercus kelloggii*). Both of these species are culturally important as well as important to wildlife and recreation and cover 79184 and 8351 acres (320 and 34 km^2^), with 24% and 12% protected, respectively. Collectively, these vegetation communities represent 30% of the study area, so provide a good representation of how overall vegetation may shift over time.

Coyote brush is native from northern Baja California, Mexico to southern Oregon. In California, this shade-intolerant evergreen shrub is widely distributed west of the coast ranges at elevations less than 2000 ft (600 m) and is closely associated with coastal scrub and chaparral plant communities. Chamise, another evergreen shrub, is native to California and Baja California. This drought-tolerant species dominates chaparral communities throughout California below 6000 ft (2000 m), often found on hot and dry south-facing slopes.

Douglas fir is native to western North America and found from southwestern British Columbia, Canada south into the central coastal and Sierra Nevada regions of California. This long-lived evergreen conifer tree is widespread in mixed conifer forests at elevations below 6000 ft (2000 m), adapted to moist mild climates, and requires well-drained soils. Coast redwood distribution is limited to a coastal strip extending fewer than 50 miles (80 km) inland from southwestern Oregon to central California. This long-lived, shade-tolerant, coniferous tree is found primarily at elevations below 2500 ft (760 m). It is adapted to the coastal climate, occurring in the fog belt and high moisture areas that buffer the effect of summer heat and drought, and is associated with acidic, well-drained soils. Coast redwoods dominate the forests they are found in and often co-occur with Douglas fir.

Coast live oak is a drought-resistant broadleaf evergreen tree which is found in the coast ranges from north central California south to Baja California up to 4500 ft (1400 m) elevation. This fire-adapted species is associated with well-drained soils and dominates lower-elevation oak woodlands. California black oak is found from southwestern Oregon to California at elevations from 650 ft (200 m) up to 8500 ft (2600 m). This highly drought tolerant deciduous tree species is most commonly associated with ponderosa pine (*Pinus ponderosa*) and mixed conifer forests but also occurs in mixed-oak woodlands.

### Modeling extents

Although our objective was to provide maps only for the GGBN region, training SDM models using data across entire species’ ranges better captures the variation of climatic conditions in which they can persist. Therefore, we expanded our model extents to the California portion of the California Floristic Province (CFP), a region for which high-resolution climate data are available. This area is almost entirely within California and contains the entirety or near entirety of the ranges of 5/6 of our study species. For the one species (Douglas fir) whose range extends beyond the CFP, the CFP contains the southernmost extent and captures a substantial amount of climatic variability within its range. We developed models for the species’ ranges within the California Floristic Province and then clipped model outputs to the extent of the study region (Fig 1). For each species of interest, we derived range maps from Jepsons ecological subregions and ranges depicted by eFlora (https://ucjeps.berkeley.edu/eflora/geography.html). We defined each species model extent as a 5 km buffer of its range as depicted by eFlora within the California Floristic Province (S1 Fig).

### Species occurrence-non-occurrence data

We obtained species data spanning 1980–2023 from California Department of Fish and Wildlife (CDFW), Consortium of California Herbaria (CCH), and Calflora. We extracted occurrence data for the species of interest from these and a previously acquired database [40] and filtered for quality by removing records that met any of the following conditions: located outside the California Floristic Province, lack date information, have low location accuracy, are suspected to be cultivated, located in areas classified as barren, cultivated crops, developed high intensity, or open water land cover [44], or are more than 5 km outside of the species’ range. Merging the remaining occurrences from our multiple datasets, each with potential spatial sampling bias, resulted in uneven point distributions with clusters that may increase spatial autocorrelation [45,46]. Spatial thinning is a widely used method to overcome sampling bias, reduce model over-fitting and autocorrelation, and increase model performance by decreasing point density in over sampled areas [47–49]. Therefore, we applied a minimum nearest neighbor distance to filter the merged occurrence data to 1-km, based on exploratory analyses and our prior modeling experience.

We derived absence data by extracting relevé surveys more than one pixel (270-m) away from occurrences for each species such that we did not have more than one occurrence or non-occurrence per cell. Relevé plant surveys provide complete lists of species occurrences per plot, thus providing records of both presence and absences for shrub and tree species. We used a similar filtering process for absences as we did for presences, where we removed records with low accuracy, falling within the land cover mask or > 5 km outside the species range. We merged remaining points and filtered those to a 1-km minimum nearest neighbor distance, leaving 749 (Douglas fir) – 3247 (California black oak) occurrences and 773 (Douglas fir) – 4231 (California black oak) non-occurrence points for modeling (S2 Table). For each species, we merged the cleaned and filtered presence and absence point layers.

### Environmental variable data

We derived 23 potential environmental variable layers including topography, soil, hydrology, and climate from best available spatial data sources (S3 Table). We resampled all variables to align with our coarsest resolution dataset, climate, at 270m, using the ArcMap ‘Resample’ tool with bilinear interpolation.

We used 30-year normal climate and hydrology variables from the California Basin Characterization Model (BCM) v6.5 dataset (a regional water balance model; [50]) for three time-periods (1921–1950, 1951–1980, and 1981–2010) to calibrate baseline models. This BCM dataset provides fine-resolution climatic water deficit and actual evapotranspiration layers relevant to plant ecology, and these variables were lacking in the most recent CMIP 6 climate datasets. This is why we used the Coupled Model Intercomparison Project, Phase 5 (CMIP5) global climate models (GCMs) for our future projections. We used 2070–2099 for future projections from three downscaled GCMs representing a range of potential future climate trajectories: CNRM-CM5 ([51]; hot/wetter), CCSM4 ([52]; warm/wet), and MIROC-ESM ([53]; hot/dry), all using the Representative Concentration Pathways' (RCP) 8.5 emissions scenario. Two of these climate models were also among the ones most highly recommended for use in the state of California [54].

We extracted all environmental variable values to the species occurrence and non-occurrence points and exported the csv tables for modeling in the R statistical program. We converted all environmental variable layers to tif raster file format for use in R (version 4.4.1, R Core Team 2024).

### Species distribution models

Extensive research documenting sources of SDM uncertainty has consistently shown the SDM modeling algorithm as the largest influence on model performance (e.g., [14,15]), with individual model types resulting in large variation in both spatial predictions (e.g., [16–18]) and model performance [19]. This makes model selection especially challenging because no one model consistently outperforms others across species or geographies [55].

In response to this variability and uncertainty produced by using different models, a number of scientists have demonstrated the benefits of using consensus methods, which combine results from several model types into an ensemble of predictions [56]. In other words, the ensemble approach involves the construction of multiple SDMs using different modeling algorithms and then synthesizing model results based on one of several approaches, such as averaging model predictions or choosing a suite of predictions based on model performance [20–22]. Studies comparing model algorithms to ensemble approaches have found the ensemble predictions to have the highest accuracy [57].

We created distribution models under baseline (historical) climate conditions using the best-performing combination of uncorrelated predictors for each species. To identify the best time period for historical climate layers (1921–1950, 1951–1980, or 1981–2010) and best performing uncorrelated predictors (Pearson correlation < 0.7) for each species, we used Welch’s t-tests to evaluate the difference in means between values at the presence and absence points [58]. We retained the most informative variables by selecting all with p-values ≤ 0.01. We selected the climate variable time-period that resulted in the highest t-value for each species. We removed any variable that had a p-value > 0.01 and retained the climate variable time-period that resulted in the highest t-value. For the shorter-lived shrubs (coyote brush and chamise), however, we used 1981–2010. For each species, we then assessed correlations among our selected variables across the California Floristic Province and removed those with the lower t-value from any group that had a Pearson correlation >= 0.7. We used the remaining variables to develop the SDMs for each species.

We used an ensemble modeling approach implemented in the flexSDM R package [22] to create baseline models for all species using the following 6 presence-absence species distribution modeling algorithms: generalized additive (GAM), generalized boosted regression (BRT), generalized linear (GLM), neural networks (ANN), random forest (RF), and support vector machine (SVM) models. To partition occurrence data and evaluate model performance, we used spatial block cross-validation, testing 30 different block sizes and dividing species data into 4 partitions to reduce spatial autocorrelation [22,40]. We created ensemble models from all individual models meeting a performance threshold of having an area under the receiver operating characteristic curve (AUC)>= 0.7. We used the weighted mean method of combining individual model outputs into a single ensemble layer based on model performance as measured with the true skill statistic (TSS) [59].

### Model evaluation

To evaluate model performance of the baseline distribution models, we used multiple threshold-dependent and independent approaches available in the flexSDM R package [22]. Threshold-dependent approaches require converting SDM continuous (0–1) probability predictions to binary outputs (predicted present/absent or suitable/unsuitable) to determine the model’s ability to differentiate between occurrences and non-occurrences. To define “suitable habitat” for threshold-dependent model evaluation methods, we used the maximum training sum of sensitivity and specificity (MAXSS), a model-specific threshold shown to optimize discrimination between presence and absence [60] to classify model outputs into suitable and unsuitable areas.

The threshold-dependent evaluation metrics we used included model sensitivity, specificity, overall accuracy, TSS, Sorensen index, and Jaccard index. Sensitivity, specificity, and overall accuracy range from 0–1 and are based on a confusion matrix comparing predicted versus observed presences and absences [61]. Sensitivity, or true-positive rate, is the proportion of correctly predicted presences and is used to measure omission error [62]. Specificity, or true-negative rate, is the proportion of correctly predicted absences, measuring the commission error [62]. Overall accuracy measures the correct classification of both presences and absences.

TSS ranges from −1–1 and integrates errors of both omission and commission. TSS was proposed as a measure unaffected by prevalence [59], but has since also been criticized for being dependent on sample prevalence, as with AUC [61]. This metric is defined as the average of the net prediction success rate for present sites and for absent sites [63].

The last two threshold-dependent evaluation metrics, the Jaccard and Sorensen Indices, are both similarity measures ranging from 0 to 1 which measure the similarity between predictions and observations [61]. These indices are widely used in community ecology analyses to evaluate community similarity.

Threshold-independent assessment methods include AUC (area under the receiver operating characteristic (ROC) curve) [64] and the continuous Boyce Index [65]. AUC, considered a measure of performance across all possible thresholds, while often criticized [66], is one of the most commonly used evaluation measures [63]. AUC ranges from 0 to 1 and represents the probability that a randomly-drawn presence site has a higher score than a randomly drawn absence site. The Boyce Index, developed for the evaluation of presence-only data models, ranges from −1–1. This metric uses the relative frequencies between presence points and background points within a series of moving window bins spanning the entire range of predicted values from the SDM [67] to create predicted-to-expected ratio curves that evaluate model robustness, discrimination, and deviation from randomness [65]. The Spearman rank correlation coefficient measures the correlation between proportion of sites in each prediction class and expected proportion of predictions in each prediction class based on the proportion of the landscape in that class.

We also evaluated how our baseline distributions agreed with a recent fine scale vegetation map produced for the region from a combination of orthophotography, lidar, and field validation [68] with a minimum mapping unit of approximately 4047 m^2^ (1 acre). These maps reflect ground conditions circa 2013 (Sonoma County) to 2018 (Marin and San Mateo County) and are intended for use at scales of up to 1:5000.

### Model projections and post processing

After developing the SDMs using the predictor maps representing recent conditions, we then applied the model algorithm to the same climatic predictor variables in the model, but swapping out the baseline data for the projections mapped under the GCMs. We projected the baseline models using future end of century (2070–2099) climate predictors under the three GCMs (CNRM-CM5, CCSM4, and MIROC-ESM). We modified the baseline and future ensemble model outputs by assigning all pixels with values less than the maximum sum of sensitivity and specificity threshold a value of 0. We used a land cover mask to convert the value of areas with high intensity development, barren, cultivated crops, and open water [44] land covers to 0. We clipped model outputs to the GGBN terrestrial boundary.

We evaluated the baseline climate niche of each species by plotting the mean values of seasonal climate variables for occurrence points across their model extents. We obtained variable importance metrics for the algorithms within FlexSDM with that option (GAM, GRT, GLM, and ANN) and ranked variables by mean importance for each species. We created partial dependence plots for the top three most important variables for each species’ GLM model as the GLM plots were smoother and easier to interpret than the other algorithms used.

To evaluate projected species distribution shifts, consensus among GCMs, and potential refugia, we overlaid and compared output maps. We calculated the percent change in climate variables relative to baseline conditions and calculated the area predicted suitable for each species under baseline and future conditions. To evaluate the directionality of potential range shifts, we calculated the mean elevation and distance to coast within suitable areas as well as the magnitude and angle of distributional changes using the SDM toolbox (v2.4; [69]).

## Results

### Baseline models

The ensemble models for all species had good to excellent performance as measured by a suite of metrics, with model performance varying by species (Table 1). Agreement with the fine-scale vegetation map ranged from 0.78 (chamise) to 0.96 (coast redwood).

**Table 1:** Model evaluation metrics.

Species	Sens. (TPR)	Spec. (TNR)	AUC	TSS	Boyce	Jaccard	Sorensen	Agreement with Fine Scale Veg Map
Chamise	0.80	0.81	0.88	0.61	0.96	0.72	0.83	0.78
Coyote Brush	0.77	0.65	0.77	0.42	0.94	0.64	0.78	0.91
Douglas Fir	0.84	0.68	0.83	0.52	0.91	0.64	0.77	0.95
Coast Live Oak	0.82	0.62	0.75	0.44	0.89	0.68	0.80	0.86
California Black Oak	0.84	0.78	0.87	0.63	0.99	0.66	0.80	0.89
Coast Redwood	0.85	0.87	0.94	0.72	0.97	0.67	0.80	0.96

The relative importance of the climate, topography, and soil variables differed by species and SDM model type (S2 Fig). For most species, only one to three variables dominated in importance. The top-ranking variable was climatic for all species, but soil characteristics were included in the top three-ranking variables for all species except the two oaks.

**Fig 2:**
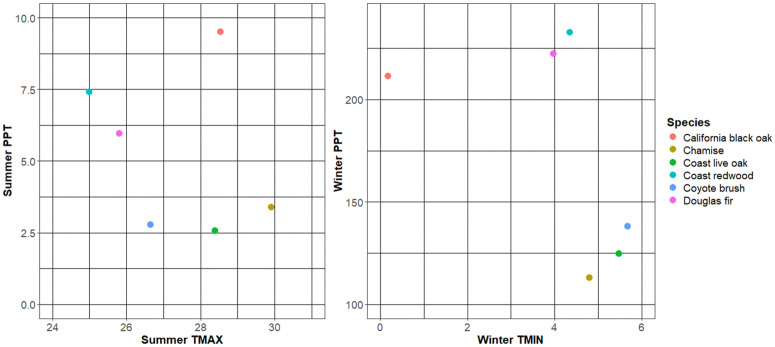
Mean summer precipitation and maximum summer temperature, and mean winter precipitation and minimum winter temperature (1981-2010) at occurrence locations throughout the model extents of six plant species.

The two shrub species, chamise and coyote brush*,* are distributed in relatively warm and dry locations (Fig 2), which is reflected in the response curves for their top-ranked predictor variables (S3 Fig). There is a positive relationship between habitat suitability and climatic water deficit for chamise and a positive relationship between habitat suitability and winter minimum temperature for coyote brush (S3 Fig). The two oak species occur in different areas climatically. Coast live oak is distributed in relatively warm and dry locations, similar to the two shrubs, and this is reflected in the response curves for the three top-ranking variables (S3 Fig), although variable importance is more evenly distributed across predictors and models relative to other species (S2 Fig). On the other hand, black oak is located in areas that are wetter, with moderate summer temperatures and cold winter temperatures, with nonlinear response curves for the top-ranking climate variables, summer precipitation, winter minimum temperature, and summer maximum temperature (Figs 2 and S3). Douglas fir occurs in areas that are relatively cool and moderately wet in the summer and warm and wet in the winter (Fig 2), and the response curves are nonlinear for the top-ranking climate variables, winter precipitation and temperature (S3 Fig). Finally, coast redwood occurs in wet locations with cool summer temperatures and warm winter temperatures,also with nonlinear response curves (Figs 2 and S3).

### Projected changes in climate predictors

**Fig 3:**
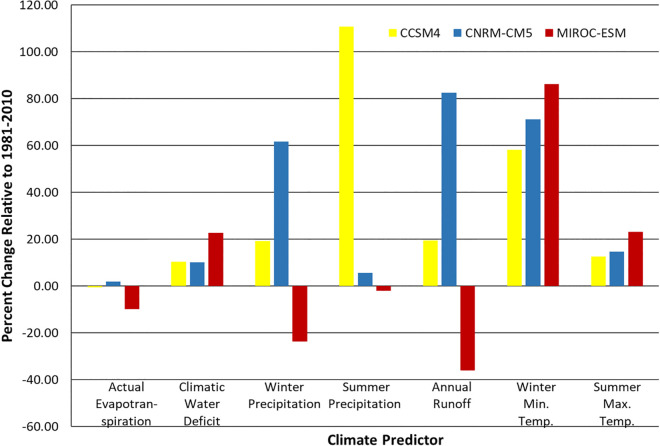
Percent change in mean value of 30-year normal climate predictors by GCM across the GGBN relative to the 1981-2010 baseline. CCSM4 in yellow, CNRM-CM5 in blue, and MIROC-ESM in red.

Changes in climate predictors relative to baseline conditions differed by variable and GCM (Fig 3). Directions of change were consistent across the three baseline time periods except for summer precipitation, which decreased relative to baseline for the older historical baseline normals (1921–1950, 1951–1980) but increased relative to the more recent baseline period (1981–2010) under CNRM-CM5 (S4 Fig).

For all three GCMs, climatic water deficit, winter minimum temperature, and summer maximum temperature were projected to increase across the GGBN. The projected increase in winter minimum temperature was much larger than the increase for minimum summer temperature and climatic water deficit, particularly under MIROC-ESM (Fig 3).

Under MIROC-ESM, both winter precipitation and annual runoff were projected to decrease by more than 20%; but under CCSM4 and CNRM-CM5, winter precipitation was projected to increase by approximately 20% and 60% respectively. In terms of summer precipitation, there was a slight decrease projected under MIROC-ESM and a slight increase under CNRM-CM5. Under CCSM4, however, summer precipitation was projected to dramatically increase by more than 100%. Actual evapotranspiration remained relatively stable under CCSM4 and CNRM-CM5 and was projected to decrease by nearly 10% under MIROC-ESM.

### Projected changes in ensemble distribution models

**Fig 4:**
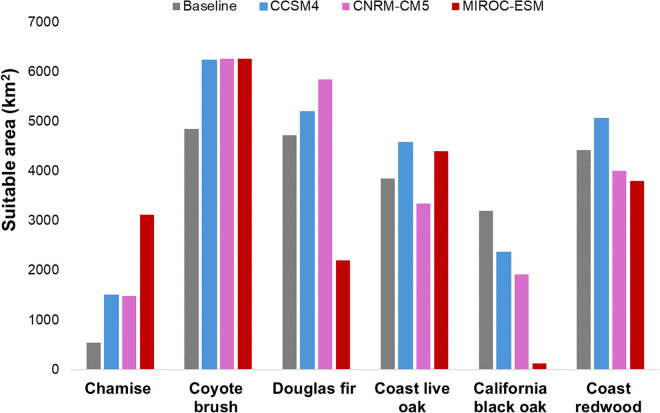
Area (km^2^) modeled and projected as suitable under baseline and future climate conditions within the GGBN region.

Within the GGBN, relative to baseline conditions, chamise and coyote brush were both projected to have large increases in modeled suitable area while a large decrease was projected for California black oak under all three GCMs (Fig 4). Directionality of projected changes in amounts of suitable area varied by GCM for the remaining species.

**Fig 5:**
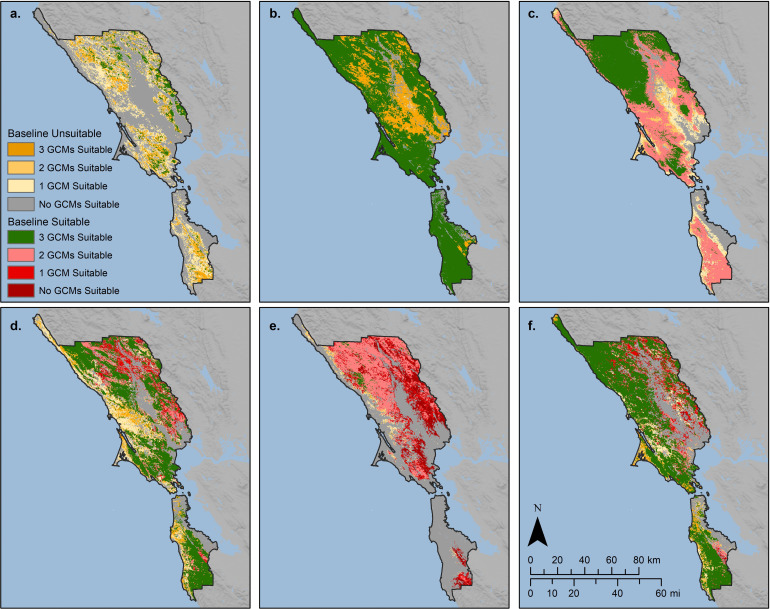
Areas likely to persist, expand, and contract within the GGBN region for a. chamise (*Adenostoma fasciculatum*), b. coyote brush (*Baccharis pilularis*), c. Douglas fir (*Pseudotsuga menziesii*), d. coast live oak (*Quercus agrifolia*), e. California black oak (*Quercus kelloggii*), and coast redwood (*Sequoia sempervirens*). Green indicates areas of persistence, orange indicates expansion, and red indicates contraction.

The chamise ensemble model projected a 173–475% increase in suitable area (Fig 4). Climatic water deficit, the most important predictor in the chamise models, was projected to increase by 10–22% (Fig 3). The modeled baseline distribution shows small patches of suitable areas largely inland, and these increase in size and contiguity under all 3 future climates, most dramatically under MIROC-ESM (S5 Fig). Our model projections show great potential for occupancy expansion in the GGBN region under all GCMs (Fig 5a).

The coyote brush model projected an approximate 50% increase in suitable area under all 3 GCMs (Fig 4). Winter minimum temperature, the most important predictor in the coyote brush models, was projected to increase from 58% to 86% across the GGBN (Fig 3). The modeled distribution of this species within the GGBN under baseline conditions is extensive (S6a Fig) and covers nearly the entire region under all 3 GCMs modeled (S6b–d Fig). Predicted suitable area for this species was projected to remain stable and likely expand (Fig 5b).

Suitable area for Douglas fir within the GGBN relative to baseline was projected to increase by 10–23% under CCSM4 and CNRM-CM5 but contract by 53% under MIROC-ESM (Fig 4), tracking projected changes in winter precipitation (Fig 3), the most important predictor for this species. The modeled baseline distribution for this species is extensive (S7a Fig), excluding lower elevations in the central north section which is filled in slightly under CCSM4 (S7b Fig) and significantly under CNRM-CM5 (S7c Fig). Under MIROC-ESM, suitable habitat strongly contracted, with remaining suitable areas confined to the northern portions of the GGBN (S7d Fig). These larger patches in the north were projected to remain stable but are mostly surrounded by predicted contraction areas (Fig 5c).

The models projected suitable area for coast live oak within the GGBN to increase from 14–19% under CCSM4 and MIROC-ESM and to decrease by 13% under CNRM-CM5 (Fig 4). This species had multiple important predictors, but patterns in projected climate changes do not obviously match projected changes in this species modeled distributions. While winter minimum and summer maximum temperature were both projected to increase from 80 to >100% and 13–24% respectively under all three GCMs, responses in summer precipitation were more varied. Under CCSM4, summer precipitation was projected to increase by nearly 90% but decrease from approximately 5–12% under CNRM-CM5 and MIROC-ESM. Discrepancy in predictions across GCMs therefore reflect different combinations of this species’ responses to different climate (and other) predictor variables relative to where and how much change is projected in the GCMs. This species baseline modeled distribution is extensive with many large contiguous patches across the GGBN (S8a Fig). The ensemble models projected large stable patches of suitable areas in a central strip running north-south, with areas of potential contraction inland north of the bay and possible expansion areas in the central coastal regions of the GGBN (Fig 5d).

Suitable area for California black oak within the GGBN is projected to contract by 25–96% (Fig 4), tracking projected changes in summer maximum and winter minimum temperatures (Fig 3), among the most important predictors for this species. The modeled baseline distribution of this species is largely confined to a few large patches in the northern portion of the GGBN (S9a Fig). These patches decrease in value and size under the 3 projections (S9b–d Fig). There is one small patch of projected stable suitable area for this species within the GGBN surrounded by large swaths of areas projected to contract (Fig 5e).

Suitable area for coast redwood within the GGBN was projected to increase by 15% under CCSM4 but contract by 9–14% under CNRM-CM5 and MIROC-ESM (Fig 4), tracking projected changes in summer precipitation (Fig 3), which, with winter minimum temperature, was the most important predictor for this species. Our baseline distribution model for coast redwood shows extensive suitable areas distributed across the GGBN (S10a Fig), with subtle areas of expansion concentrated along coastal zones under CCSM4 (S10b Fig), and significant decreases in values and extent under CNRM-CM5 (S10c Fig) and MIROC-ESM (S10d Fig). Large patches of stable suitable areas were projected along the coast, with small patches of potential contraction areas inland (Fig 5f).

### Direction of distribution changes

Suitable habitat for four of the priority species, including the two shrub species, chamise and coyote brush, as well as coast live oak and coast redwood, was projected to move on average to lower elevations in the reserve (S4 Table). Habitat for Douglas fir was also projected to shift to lower elevations for two of the three climate scenarios (CCSM and CNRM) with a projected shift to higher elevations under MIROC. For black oak, habitat was projected to primarily shift to higher elevations in the reserve. For four of the species, including chamise, coast live oak, coast redwood, and black oak, projected habitat shifted closer to the coastline, whereas coyote brush was projected to shift farther inland (S5 Table). There was little shift relative to the coast for Douglas fir.

In terms of latitude, the habitat for chamise, coast live oak, and coast redwood was projected to shift southward toward the equator (S6 Table). For coyote brush and black oak, the directionality of habitat was projected to shift to the northwest. The latitudinal direction of habitat shift for Douglas fir varied according to climate scenario.

## Discussion

Given rapid global change and its threats to biodiversity, conservation planners and decision-makers increasingly need to anticipate how, when, and where ecosystems are most likely to change. Although many species face multiple interacting threats, changes in climate alone are likely to result in dramatic shifts in the potential places where biota can establish and persist in the future, particularly in reserves where land is protected for conservation. While more complicated modeling approaches can estimate the effects of multiple threats, SDM is the dominant approach available for assessing species’ habitat suitability under future climates.

This study presents a set of lessons learned after applying carefully constructed ensemble SDMs to six dominant plant species in a small biodiversity reserve. Rather than offering new principles about species’ response to climate change, these lessons illustrate how SDM approaches can be operationalized to support decision-making and conservation planning in spatially-constrained management contexts.

Practitioners generally recognize that SDMs are not deterministic forecasts, but tools that are integrated with local ecological knowledge, monitoring data, and management experience to inform decision-making. In this context, the models presented here fill an important data gap by providing spatially explicit information on potential future habitat suitability, helping to inform discussions around restoration prioritization, resistance versus adaptation strategies, and long-term planning under uncertainty.

### Species’ habitat may either expand or contract in response to climate change, and this varies according to individual species

One of the biggest challenges in understanding how biodiversity may be impacted by global change is that SDMs are typically developed for individual species, particularly in applied planning contexts, although multi-species approaches such as stacked SDMs, joint SDMs, and community-level models are increasingly used to evaluate community-wide responses (e.g., [70–72]). Yet climate change has the potential to affect all species. While species’ traits may be useful in identifying functional groups that respond similarly to climate change or other threats like disturbance [73–75], our results underlined how habitat shifts and response curves to climate variables are nevertheless individualistic, even for the two species within the same genus (i.e., *Quercus*). Thus, consistent with other studies, climate change may either expand or decrease plant habitat suitability, and the outcome is mostly species-specific, although other studies have found that factors such as range size or dispersal distance may be associated with these differences [27,35,76]. In the GGBN, climate changes will likely favor both of the shrub species and will threaten black oak, with an uncertain prognosis for the other three species, with two of the three GCMs projecting expansion for coast live oak and Douglas fir, and two of three GCMs projecting habitat decline for coast redwood.

Some scientists account for individual species’ response to climate change by modeling thousands of different species and comparing or overlaying mapped projections (e.g., [77–79]), and others have attempted to model the distribution of vegetation types or communities [80–82]. By choosing six specific species for this study, the conservation planners at the GGBN followed the approach by [33], who, instead of directly modeling vegetation types, they created SDMs for key species that define those types. This allowed the GGBN to integrate SDM results into climate change response scenario planning. This includes incorporating findings from SDMs into holistic approaches to climate adaptation that look to expand habitat, consider assisted dispersal, or protect refugia for key species while simultaneously addressing vulnerabilities for infrastructure, cultural resources, and adjacent communities.

### Projected changes in habitat suitability reflect climatic and non-climatic factors

For all species, climate variables represented at least one of the top three most influential predictors of habitat suitability. This important role of climate in limiting species distributions [6] explains many of the projected habitat suitability changes in our results. For example, chamise is positively associated with climatic water deficit and is projected to undergo widespread expansion under all three GCMs, as climatic water deficit levels were projected to increase across the board. Coyote brush is positively associated with winter minimum temperature and this is reflected in the widespread projections of habitat increase under all three GCMs. California black oak, associated with moderate summer and winter temperatures, was projected to have its distribution contract substantially as seasonal temperatures are projected to increase under all three GCMs. In a similar geography, Ackerly [33], projected shrubland and oak woodland species to expand with hotter and drier conditions while grasslands and conifer forests were projected to decline. Coast live oak, the oak woodland species modeled here, was projected to expand with two of three GCMs, but the other oak we modeled, black oak, is often distributed at higher elevations across the state and is a deciduous species [83].

There is greater uncertainty surrounding the potential fates within the GGBN of the species with varied responses to the three GCMs evaluated. The GCMs selected for this study all project temperature warming with differences in the magnitude. However, precipitation is much more variable. Climate model projections of changes in future rainfall amounts and patterns have consistently been more variable and uncertain than temperature projections, particularly in California [54,84]. This variability may explain the discrepancies in habitat suitability for coast live oak and Douglas fir, both of which responded strongly to seasonal precipitation (summer for coast live oak and winter for Douglas fir). There were also discrepancies in habitat suitability for coast redwood, with two of the three models projecting it to decline. While precipitation was not one of the most important explanatory variables for this species (it was dominated by minimum winter temperature), Ackerly et al. [33] found that vegetation closer to the California coast was disproportionately sensitive to climate change. Thus, the differences in degree of warming among the GCMs may explain why their projections resulted in different scenarios for this species. We also did not explicitly model coastal fog, which is an important climatic mediator for this species, although the redwood results did suggest a loss of suitable habitat at the eastern edge of the current climate envelope where there is currently the least fog. Although fog was not directly modeled, variables such as summer temperature and climatic water deficit may partially capture its effect. While this uncertainty complicates projections, there are areas of agreement where species are projected to remain stable under all three GCMs. Focusing on these areas where there is more confidence in predictions may be important to consider for prioritizing future conservation management actions.

Another factor explaining future habitat suitability is the importance of additional variables, like soil or topography, that significantly limit where a species may persist. For example, Franklin et al. [82] found that soil moisture and texture altered the projected distributions of plant species in California created using only climate-based SDMs, particularly in heterogeneous landscapes. Other studies have also shown that soil and other non-climatic variables can mediate how species respond to climate change (e.g., [85,86]). While climate was often more important than soil variables for the species modeled here, both soil and topographic variables were frequently retained among the most influential predictors of habitat suitability.

### Species’ range changes may not always be upwards in elevation or polewards in latitude

While GCM predictions of precipitation remain relatively uncertain, predictions of temperature vary only in terms of degree and seasonality of warming [87]. Given the unequivocal agreement that temperatures will rise, a long-held assumption is that species’ suitable habitat will move to cooler places on the landscape, i.e., higher in elevation or polewards in latitude. Although results of many studies are consistent with this hypothesis (e.g., [34,35,88]), our results suggest variability in the direction and elevation of projected habitat change. In fact, projected habitat suitability for four of the six species was projected to shift to lower elevations on average, with another species, Douglas fir, projected to shift to lower elevations in two of the three climate scenarios. Three of the six species were also projected to shift towards the equator instead of the pole.

These results are consistent with another study in California, examining early signs of plant species’ regeneration patterns that showed species’ are not always tracking shifts in climate space or following a predictable higher elevation and latitude directional shift [89]. Ackerly et al. [33] also found that habitat expansion under warming climates was generally closer to the coast, which tends to be cooler during summer months, and at lower elevations; but species with habitat contraction were more likely to shift upwards in elevation. In California, temperature gradients shift seasonally with winter temperatures warmer toward the coast, often at lower elevations, and summer temperatures warmer inland, often higher-elevation locations. Given species’ differences in tolerance to seasonal variation in climate, these gradient shifts may explain the diverse directions of projected habitat shifts.

Another important factor to consider is the high potential for habitat refugia in topographically complex terrain, which may allow species to persist in areas at lower-elevations or latitudes [82,90,91]. Finally, there is some evidence to suggest that coastal fog in California may help to offset climate warming by reducing temperature and providing a source of moisture [92,93]. Although we did not explicitly model fog, and the future of fog may be uncertain, the effect may have been indirectly accounted for in the species’ observation data.

### SDMs can provide a baseline for conservation decisions, but habitat changes within protected areas may not be representative of other parts of species’ ranges

Consistent with the findings of others, our SDMs illustrated how the habitats of different species are likely to reassemble in the future relative to existing protected areas [28]. The SDMs developed here, using a diverse range of modeling algorithms (i.e., model ensembles) and GCMs that bracket the range of variability in projected climate futures in California, provide a relatively robust assessment of where several of the most important plant species in the GGBN may persist in or disperse to in the future. The species represent different life forms (trees and shrubs), hardwood and conifer species, and species with narrow (redwood) versus large (Douglas fir) range sizes.

The SDMs have been serving as a baseline for conservation decision-making. For example, conservation planners at GGBN have integrated the projected SDM outputs with an existing fine-scale vegetation map to identify and evaluate potential refugia availability for vegetation alliances centered around the dominant species. They defined refugia as areas where at least one GCM predicted future suitability with at least 90% overlap with a polygon of the associated mapped alliance and calculated the refugia area for each alliance of interest by land ownership and county. They further defined refugia quality as low, moderate, or high according to the number of GCMs with predicted suitability. GGBN land managers have access to these integrated datasets via an online mapping portal, which they may use to visualize where future potential suitable areas overlap with current mapped distributions [68].

Although the SDMs in this study are assisting planning efforts within the boundaries of the GGBN, one challenge in biodiversity conservation is that conservation managers within small reserves are only able to manage for the parts of species’ ranges that overlap reserve boundaries. While all species are native to California, their range sizes vary, and they may have differing prognoses in other parts of their range. For example, black oak is projected to contract substantially within the GGBN and will be a candidate for careful conservation planning and management to ensure its persistence within the region. Despite the poor prognosis for black oak habitat in the GGBN, however, other scientists have projected an increase in climatically suitable habitat for this species in parts of its range in Oregon [88]. These results underscore the value in continuing dialog and information sharing between land managers and ecoregions through partnerships such as the California Landscape Stewardship Network (https://calandscapestewardshipnetwork.org/), to maximize the impact of climate adaptation efforts and identify areas of shared conservation interest.

### Other considerations

As mentioned previously, other global change factors, such as land use change, altered disturbance regimes, or competition with other species (e.g., biotic interactions or pests) are occurring to differing degrees alongside and in combination with climate change [94]. While our SDMs estimate potential habitat suitability, they do not explicitly account for biotic interactions or dispersal limitations, which can play a stronger role in shaping species distributions and abundance at finer spatial scales, especially for recruitment and establishment phases [2,6,11,95]. In addition, although several species in the GGBN are likely to require little direct conservation action to persist under future climate conditions, changing fire regimes in the region [96] may interact with climate to alter trajectories of species’ persistence. To illustrate, while chamise is projected to experience an expansion in suitable habitat, it is particularly sensitive to short intervals between fires [97–99]. Incorporating wildfire dynamics into future modeling efforts, or addressing fire explicitly through management, may be necessary for some species.

We also note that we used CMIP5 climate projections, rather than the more recent CMIP6 projections, due to the availability of plant-relevant hydrological variables from the Basin Characterization Model. Accordingly, the primary aim of this analysis was to explore relative changes and differences among scenarios, rather than to provide precise forecasts of realized future species distributions. Because species presence and absence data are typically collected based on adult plants, SDMs trained on these data may overestimate suitability for establishment. This is because environmental conditions suitable for juveniles to survive tends to be narrower than their conspecific adults [100], and thus, young life stages are often more vulnerable to climatic controls than adults [101]. Because SDMs also do not account for species’ demographic processes like dispersal, fecundity, or carrying capacity, managers could analyze mapped output to see if future projected habitat is within species’ dispersal distances.

Finally, as is common and accepted practice in SDMs, we used 30-year climate normals as predictors, as these are most appropriate for estimating long-term change over broad scales [7,19,102]. Nevertheless, at local scales, short-term weather events, climate variability, or extended periods of drought, for example, may strongly impact population dynamics and local distributions [103]. Using shorter-term climate data has been shown to improve some SDMs [102,104–106]. However, using finer-scale temporal resolution may introduce additional uncertainty and may be more relevant for shorter-lived species than the plants considered here.

## Supporting information

S1 Fig Presence and absence localities and extents used for distribution models for a. chamise (*Adenostoma fasciculatum*), b. coyote brush (Baccharis pilularis), c. Douglas fir (*Pseudotsuga menziesii*), d. coast live oak (*Quercus agrifolia*), e. California black oak (*Quercus kelloggii*), and f. coast redwood (*Sequoia sempervirens*). (TIF)

S2 Fig Predictor importance by model algorithm for (a) chamise (*Adenostoma fasciculatum*), (b) coyote brush (*Baccharis pilularis*), (c) Douglas fir (*Pseudotsuga menziesii*), (d) coast live oak (*Quercus agrifolia*), (e) California black oak (*Quercus kelloggii*), and (f) coast redwood (*Sequoia sempervirens*). Note that importance values were not provided for BRT or SVM algorithms.
(TIF)

S3 Fig Partial dependence plots showing marginal effects of the top three mean most important variables on suitability for GLM models of (a) climatic water deficit, soil pH, and topographic wetness index for chamise (*Adenostoma fasciculatum*), (b) soil pH, summer precipitation, and winter minimum temperature for coyote brush (*Baccharis pilularis*), (c) percent clay, winter precipitation, and winter minimum temperature for Douglas fir (*Pseudotsuga menziesii*), (d) summer precipitation, winter minimum temperature, and summer maximum temperature for coast live oak (*Quercus agrifolia*), (e) summer precipitation, winter minimum temperature, and summer maximum temperature for California black oak (*Quercus kelloggii*) and (f) percent clay, summer precipitation, and winter minimum temperature for coast redwood (*Sequoia sempervirens*). (TIF)

S4 Fig Percent change in mean value of 30-year normal climate predictors by GCM across the GGBN relative to the (a) 1921–1950 and (b) 1951–1980 baseline. CCSM4 in yellow, CNRM-CM5 in blue, and MIROC-ESM in red.
(TIF)

S5 Fig Modeled and projected chamise (*Adenostoma fasciculatum*) distribution within the GGBN region under a. baseline conditions, b. CCSM4, c. CNRM-CM5, and d. MIROC-ESM. (TIF)

S6 Fig Modeled and projected coyote brush (*Baccharis pilularis*) distribution within the GGBN region under a. baseline conditions, b. CCSM4, c. CNRM-CM5, and d. MIROC-ESM. (TIF)

S7 Fig Modeled and projected Douglas fir (*Pseudotsuga menziesii*) distribution within the GGBN region under a. baseline conditions, b. CCSM4, c. CNRM-CM5, and d. MIROC-ESM. (TIF)

S8 Fig Modeled and projected coast live oak (*Quercus agrifolia*) distribution within the GGBN region under a. baseline conditions, b. CCSM4, c. CNRM-CM5, and d. MIROC-ESM. (TIF)

S9 Fig Modeled and projected California black oak (*Quercus kelloggii*) distribution within the GGBN region under a. baseline conditions, b. CCSM4, c. CNRM-CM5, and d. MIROC-ESM. (TIF)

S10 Fig Modeled and projected coast redwood (*Sequoia sempervirens*) distribution within the GGBN region under a. baseline conditions, b. CCSM4, c. CNRM-CM5, and d. MIROC-ESM. (TIF)

S1 Table Acreages for selected priority plant species across the GGBN. Source: existing fine scale vegetation map data for the GGBN region developed by Tukman Geospatial et. al (2024) and consistent with California Fish and Wildlife (CDFW) Vegetation Classification and Mapping Program (VegCAMP), the Manual of California Vegetation, and the US National Vegetation Classification (NVC). Fine scale vegetation map data depicts vegetation communities generally at the Alliance level of the NVC, and individual species selected for SDM are dominant species within each Alliance. Total area (US survey acres) for each key species was calculated in ArcGIS Pro for both the terrestrial portion of the GGBN and, using the California Protected Areas Database (CPAD 2024), for the total area of each species on protected lands.
(DOCX)

S2 Table Number of occurrence and non-occurrence points used in modeling by species. (DOCX)

S3 Table Model predictors, sources, and resolutions. (DOCX)

S4 Table Mean elevation (m) of suitable areas within GGBN. (DOCX)

S5 Table Mean distance (m) from the coast of suitable areas within GGBN. (DOCX)

S6 Table Direction of distributional shift within GGBN by GCM. The angle ranges from −180° to 180°, with 0° to the north, 90° to the east, 180° (or −180°) to the south, and −90° to the west.
(DOCX)

S7 Table Details about SDM overview, data, modeling, and predictions following the ODMAP (Overview, Data, Model, Assessment and Prediction) protocol (Zurell et al. 2020). (DOCX)
