# Supplementary material for: Lessons learned using species’ distribution models for conservation planning in the Golden Gate Biosphere reserve
Source: PLoS One. 2026 Mar 11;21(3):e0343037. doi: 10.1371/journal.pone.0343037 (PMC12978446; doi:10.1371/journal.pone.0343037)
Supplement: S1 Table — Source: existing fine scale vegetation map data for the GGBN region developed by Tukman Geospatial et. al (2024) and consistent with California Fish and Wildlife (CDFW) Vegetation Classification and Mapping Program (VegCAMP), the Manual of California Vegetation, and the US National Vegetation Classification (NVC). Fine scale vegetation map data depicts vegetation communities generally at the Alliance level of the NVC, and individual species selected for SDM are dominant species within each Alliance. Total area (US survey acres) for each key species was calculated in ArcGIS Pro for both the terrestrial portion of the GGBN and, using the California Protected Areas Database (CPAD 2024), for the total area of each species on protected lands. (DOCX) [file pone.0343037.s011.docx]

**S1 Table. Acreages for selected priority plant species across the GGBN.** Source: existing fine scale vegetation map data for the GGBN region developed by [Tukman Geospatial et. al (2024)](https://vegmap.press/gg_biosphere_veg_map_datasheet) and consistent with California Fish and Wildlife (CDFW) Vegetation Classification and Mapping Program (VegCAMP), the Manual of California Vegetation, and the US National Vegetation Classification (NVC).  Fine scale vegetation map data depicts vegetation communities generally at the Alliance level of the NVC, and individual species selected for SDM are dominant species within each Alliance.  Total area (US survey acres) for each key species was calculated in ArcGIS Pro for both the terrestrial portion of the GGBN and, using the California Protected Areas Database ([CPAD 2024](https://calands.org/cpad/)), for the total area of each species on protected lands.

| Species (Fine Scale Vegetation Map Class) | Total Area (km^2^) | Total Area (km^2^) on Protected Lands | Percent Protected |
| --- | --- | --- | --- |
| *Adenostoma fasciculatum* Alliance | 63 | 15 | 23 |
| *Baccharis pilularis* Alliance | 352 | 196 | 56 |
| *Pseudotsuga menziesii* – *Notholithocarpus densiflorus* / *Vaccinium ovatum* Association | 641 | 215 | 34 |
| *Quercus agrifolia* Alliance | 320 | 76 | 24 |
| *Quercus kelloggii* Alliance | 34 | 4 | 12 |
| *Sequoia sempervirens* Alliance | 658 | 195 | 30 |
