# Supplementary material for: Lessons learned using species’ distribution models for conservation planning in the Golden Gate Biosphere reserve
Source: PLoS One. 2026 Mar 11;21(3):e0343037. doi: 10.1371/journal.pone.0343037 (PMC12978446; doi:10.1371/journal.pone.0343037)
Supplement: S2 Table — (DOCX) [file pone.0343037.s012.docx]

**S2 Table. Number of occurrence and non-occurrence points used in modeling by species.**

| Species | Occurrences | Non-Occurrences |
| --- | --- | --- |
| Chamise | 2097 | 1243 |
| Coyote brush | 1859 | 983 |
| Douglas fir | 749 | 773 |
| Coast live oak | 2706 | 1457 |
| California black oak | 3247 | 4231 |
| Coast redwood | 862 | 1892 |
