# Supplementary material for: Lessons learned using species’ distribution models for conservation planning in the Golden Gate Biosphere reserve
Source: PLoS One. 2026 Mar 11;21(3):e0343037. doi: 10.1371/journal.pone.0343037 (PMC12978446; doi:10.1371/journal.pone.0343037)
Supplement: S3 Table — (DOCX) [file pone.0343037.s013.docx]

**S3 Table. Model predictors, sources, and resolutions.**

| **Type** | **Predictor** | **Source** | **Resolu-tion** |
| --- | --- | --- | --- |
| Climate and Hydrology (Historic 30-year Summaries: 1921-1950, 1951-1980, 1981-2010) | Annual Precipitation (mm) | 2014 California Basin Characterization Model (BCM)  (<https://geo.pointblue.org/commonsmap/index.php?ds=1129>) | 270-m |
|  | Summer Precipitation (PPT_JJA; mm) |  |  |
|  | Winter Precipitation (PPT_DJF; mm) |  |  |
|  | Summer Maximum Temperature (TMAX_JJA; ◦C) |  |  |
|  | Winter Minimum Temperature (TMIN_DJF; ◦C) |  |  |
|  | Climatic Water Deficit (CWD; mm) |  |  |
|  | Actual Evapotranspiration (AET; mm) |  |  |
|  | Annual Runoff (RUN; mm) |  |  |
| Topography | Slope (%) | USGS LANDFIRE Topographic Product (<https://landfire.gov/topographic.php>) | 30-m |
|  | Southwest Index | Derived by CBI from USGS LANDFIRE Topographic Products (<https://landfire.gov/topographic.php>) | 30-m |
|  | Solar Insolation Index |  |  |
|  | Topographic Wetness Index |  |  |
|  | Heat Load index |  |  |
|  | Terrain Ruggedness | USGS – Welty and Jeffries 2018. (<https://www.sciencebase.gov/catalog/item/5ab296d2e4b081f61ab4601a>) | 30-m |
|  | Topographic Heterogeneity | NatureServe | 90-m |
| Soils | Soil Thickness | Gridded National Soil Survey Geographic Database (gNATSGO), USDA NRCS (<https://www.nrcs.usda.gov/resources/data-and-reports/gridded-national-soil-survey-geographic-database-gnatsgo>) | 10-m |
|  | Soil pH |  |  |
|  | Available Water Capacity |  |  |
|  | Percent Clay |  |  |
|  | Percent Sand |  |  |
|  | Soil Porosity | California Basin Characterization Model (BCM) v8 Input (<https://www.sciencebase.gov/catalog/item/5ff8e4f9d34e52c3b3d9d53a>) | 270-m |
|  | Productivity Index | USDA U.S. Forest Service  (<https://www.fs.usda.gov/foresthealth/applied-sciences/mapping-reporting/soil-drainage.shtml>) | 240-m |
|  | Drainage Index |  |  |
