# Supplementary material for: Lessons learned using species’ distribution models for conservation planning in the Golden Gate Biosphere reserve
Source: PLoS One. 2026 Mar 11;21(3):e0343037. doi: 10.1371/journal.pone.0343037 (PMC12978446; doi:10.1371/journal.pone.0343037)
Supplement: S4 Table — (DOCX) [file pone.0343037.s014.docx]

**S4 Table. Mean elevation (m) of suitable areas within GGBN.**

| Species | Baseline | CCSM | CNRM | MIROC |
| --- | --- | --- | --- | --- |
| Chamise | 314.62 | 287.45 | 287.08 | 264.57 |
| Coyote Brush | 217.73 | 207.77 | 207.09 | 206.90 |
| Douglas Fir | 247.48 | 226.29 | 221.53 | 232.38 |
| Coast Live Oak | 272.02 | 272.48 | 296.66 | 363.35 |
| California Black Oak | 254.84 | 238.80 | 219.48 | 330.81 |
| Coast Redwood | 239.59 | 225.58 | 223.60 | 234.68 |
