# Supplementary material for: Lessons learned using species’ distribution models for conservation planning in the Golden Gate Biosphere reserve
Source: PLoS One. 2026 Mar 11;21(3):e0343037. doi: 10.1371/journal.pone.0343037 (PMC12978446; doi:10.1371/journal.pone.0343037)
Supplement: S5 Table — (DOCX) [file pone.0343037.s015.docx]

**S5 Table. Mean distance (m) from the coast of suitable areas within GGBN.**

| Species | Baseline | CCSM | CNRM | MIROC |
| --- | --- | --- | --- | --- |
| Chamise | 29,432 | 22,934 | 22,979 | 19,870 |
| Coyote Brush | 17,132 | 18,749 | 18,779 | 18,786 |
| Douglas Fir | 21,886 | 20,225 | 18,630 | 15,676 |
| Coast Live Oak | 25,321 | 22,974 | 21,263 | 10,915 |
| California Black Oak | 18,986 | 18,818 | 18,747 | 19,535 |
| Coast Redwood | 18,054 | 16,779 | 14,833 | 14,146 |
