# Supplementary material for: Lessons learned using species’ distribution models for conservation planning in the Golden Gate Biosphere reserve
Source: PLoS One. 2026 Mar 11;21(3):e0343037. doi: 10.1371/journal.pone.0343037 (PMC12978446; doi:10.1371/journal.pone.0343037)
Supplement: S6 Table — The angle ranges from −180° to 180°, with 0° to the north, 90° to the east, 180° (or −180°) to the south, and −90° to the west. (DOCX) [file pone.0343037.s016.docx]

**S6 Table. Direction of distributional shift within GGBN by GCM.** The angle ranges from -180° to 180°, with 0° to the north, 90° to the east, 180° (or -180°) to the south, and -90° to the west.

| Species | CCSM | CNRM | MIROC |
| --- | --- | --- | --- |
| Chamise | -173.53 | -178.43 | -169.22 |
| Coyote Brush | -7.57 | -7.57 | -7.57 |
| Douglas Fir | -168.74 | 171.45 | -150.23 |
| Coast Live Oak | -49.98 | -47.99 | -63.70 |
| California Black Oak | 157.91 | 150.90 | -31.04 |
| Coast Redwood | 163.90 | 173.16 | -165.11 |
