# Supplementary material for: Lessons learned using species’ distribution models for conservation planning in the Golden Gate Biosphere reserve
Source: PLoS One. 2026 Mar 11;21(3):e0343037. doi: 10.1371/journal.pone.0343037 (PMC12978446; doi:10.1371/journal.pone.0343037)
Supplement: S7 Table — (DOCX) [file pone.0343037.s017.docx]

**S7 Table.**  **Details about SDM overview, data, modeling, and predictions following the ODMAP (Overview, Data, Model, Assessment and Prediction) protocol (Zurell et al. 2020).**

| **ODMAP element** | **Contents** |
| --- | --- |
| Overview |  |
| Authorship | **Authors:** Alexandra D Syphard, Heather Rustigian-Romsos, Daniel Franco, and Alison Forrestel  **Contact email:** asyphard@consbio.org  **Title:** Lessons learned using species’ distribution models for conservation planning in the Golden Gate Biosphere reserve |
| Model objective | **SDM objective:** Mapping currently suitable habitat and predicting future habitat suitability under climate change to evaluate how climate change projections may alter the distribution and occurrence of six priority plant species within the Golden Gate Biosphere Network (GGBN) to help managers prioritize locations for conservation and management action to protect potential refugia  **Main target output:** maps of probability of species presence under current and projected future conditions |
| Taxon | 6 priority plant species (2 shrubs, 4 trees) within the Golden Gate Biodiversity Network, all native to the California Floristic Province |
| Location | California Floristic Province, California, USA |
| Scale of analysis | Spatial extent (Lon/Lat):  California Floristic Province: Longitude 124.41° W-116.05° W, Latitude 32.53° N – 42.01° N  Spatial resolution: 270m  Temporal extent: Climate normals (30-year average for 1921-1950, 1951-1980, and 1981-2010) used for calibrating models and delineating currently occupied habitat  Temporal projection: 30-year average for 2070-2099 used for model projections and estimating suitability change |
| Biodiversity data overview | Observation type: field surveys compiled from multiple agencies and organizations  Response type: presence/absence |
| Type of predictors | Climatic, hydrologic, edaphic, and topographic |
| Conceptual model/hypotheses | Tree and shrub distributions are influenced by climate, hydrology, soils, and topography and may shift with climatological changes |
| Assumptions | We assumed that the species-environment relationship was in equilibrium, that all relevant predictor variables were included in our models, and that the full range of climatic variation within each species range was captured |
| SDM algorithms | **Model algorithms:** generalized additive (GAM), generalized boosted regression (BRT), generalized linear (GLM), neural networks (ANN), random forest (RF), and support vector machine (SVM)  **Model averaging:** Ensemble models were created from all individual models with AUC >= 0.7 using the weighted mean method based on model performance as measured with the true skill statistic (TSS) |
| Model workflow | *flexsdm* package (https://github.com/sjevelazco/flexsdm) |
| Software | R statistical program |
| Data |  |
| Biodiversity data | **Taxon**: Coyote brush (*Baccharis pilularis*), chamise (*Adenostoma fasciculatum*), coast redwood (*Sequoia sempervirens*) Douglas fir (*Pseudotsuga menziesii*), coast live oak (*Quercus agrifolia*) and California black oak (*Quercus kelloggii*)  **Taxonomic reference system**: Jepson Flora of California  **Ecological level**: species  **Data sources**: Vegetation survey data (1980-2023) compiled by California Department of Fish and other sources with both rapid and relevé surveys, combined with opportunistic data from the Consortium of California Herbaria and Calflora.  **Sample size per taxon:**  *Baccharis pilularis*: 1859P/983A  *Adenostoma fasciculatum*: 2097P/1243A  *Sequoia sempervirens*: 862P/1892A  *Pseudotsuga menziesii*: 749P/773A  *Quercus agrifolia*: 2706P/1457A  *Quercus kelloggii*: 3247P/4231A  **Data cleaning/filtering:** Data were cleaned by removing records that met any of the following conditions: lacked date information, had low location accuracy, were suspected to be cultivated or were located in areas classified as barren, cultivated crops, developed high intensity, or open water land cover. Data were filtered using 1-km minimum nearest neighbor distance.  **Absence data collection:** Relevé plant surveys provide complete lists of species occurrences per plot, thus providing reliable records of both presence and absences for shrub and tree species.  **Potential errors and biases:** Some species included have geographic distributions that extend beyond California. Because of climate data availability limitations, we only included occurrences within the state of California and we acknowledge that this may affect estimates of species environmental niches. |
| Data Partitioning | Spatial block cross-validation was used to partition occurrence data, testing 30 different block grid-sizes, dividing species presence and absence records into four spatially structured partitions that reduce spatial autocorrelation. |
| Predictor variables | **Predictor variables, sources, resolutions, time periods, and extents:**  Climate and hydrology: climatic water deficit, actual evapotranspiration, minimum winter temperature, maximum summer temperature, annual, summer, and winter precipitation, and annual runoff (2014 California Basin Characterization Model (BCM, <http://climate.calcommons.org/dataset/2014-CA-BCM>), 270m, 1921-1950, 1951-1980, 1981-2010, California)  Topography: slope (USGS LANDFIRE Topographic Product (<https://landfire.gov/topographic.php>), 30m, CONUS), southwest index, solar insolation index, topographic wetness index, and heat load index (derived from USGS LANDFIRE Topographic Products (<https://landfire.gov/topographic.php>, 30m, CONUS), terrain ruggedness (USGS – Welty and Jeffries 2018 (<https://www.sciencebase.gov/catalog/item/5ab296d2e4b081f61ab4601a>), 30m, Western US), and topographic heterogeneity (NatureServe, 90m, Western US)  Soils: thickness, soil pH, available water holding capacity, percent clay, and percent sand (Gridded National Soil Survey Geographic Database (gNATSGO), USDA NRCS (<https://www.nrcs.usda.gov/resources/data-and-reports/gridded-national-soil-survey-geographic-database-gnatsgo>), 10m, CONUS), porosity (California Basin Characterization Model (BCM) v8 Input (<https://www.sciencebase.gov/catalog/item/5ff8e4f9d34e52c3b3d9d53a>), 270m, California), productivity index and drainage index (USDA U.S. Forest Service  (<https://www.fs.usda.gov/foresthealth/applied-sciences/mapping-reporting/soil-drainage.shtml>), 240m, CONUS)  **Projection:** NAD83 / California Albers  **Data processing:** Where necessary, all datasets were resampled to 270m, and clipped to the study area extent. |
| Transfer data for projection | **Climate models and data sources:** Climate normals (2070-2099) from three downscaled Coupled Model Intercomparison Project, Phase 5 (CMIP5) global climate models (CNRM-CM5, CCSM4, and MIROC-ESM), using the Representative Concentration Pathways' (RCP) 8.5 emissions scenario were used to project the SDMs onto a range of potential climate trajectories (2014 California Basin Characterization Model (BCM, <http://climate.calcommons.org/dataset/2014-CA-BCM>), 270m, 1921-1950, 1951-1980, 1981-2010, California, NAD83 / California Albers projection) |
| Model |  |
| Variable pre-selection | Based on previous experience, ecologically relevant potential environmental predictor variables (N=23) were selected from best available data sources including topography, soil, hydrology, and climate. |
| Multicollinearity | Distribution models under baseline (historical) climate conditions were created using the best-performing combination of uncorrelated predictors for each species. We used Welch’s t-tests to evaluate the differences in means between values at presence and absence points to identify the best time-period for historical climate layers and best performing predictors for each species. We retained the most informative variables by selecting those with the highest t-value for each species. We assessed correlations among our selected variables and removed those with the lower t-value from any group with a Pearson correlation >= 0.7. |
| Model settings | ANN: size = (2:(number of env. predictors)),  decay = c(seq(0.01, 1, 0.05), 1, 3, 4, 5, 10)  BRT: n.trees = seq(10, 200, 30),  shrinkage = seq(0.1, 2, 0.5),  n.minobsinnode = seq(1, 15 , 3)  RF: mtry = seq(1, number of env. predictors), 1))  SVM: C = seq(2, 80, 20),  sigma = seq(0.001, 0.2, 0.05) |
| Threshold selection | The best hyperparameter values were based on the True Skill Statistic (TSS) and the threshold that maximizes model sensitivity and specificity.  For threshold-dependent model evaluation methods, the maximum training sum of sensitivity and specificity (MAXSS) was used. |
| Model averaging/Ensembles | Ensemble models were created from all individual models with AUC >= 0.7. The weighted mean method of combining individual model outputs into a single ensemble layer based on model performance as measured with the true skill statistic (TSS) was used. |
| Assessment |  |
| Performance statistic | Model performance was evaluated using multiple statistics: sensitivity, specificity, overall accuracy, true skill statistic (TSS), Sorensen index, Jaccard index, AUC, and the continuous Boyce Index |
| Plausibility check | All coauthors reviewed the maps of current suitable conditions to assess the plausibility of the spatial projections. They also were compared to a regional high spatial resolution vegetation community map created from a combination of orthophotography, lidar, and field validations. |
| Prediction |  |
| Prediction output | **Prediction unit:** probability of species presence/habitat suitability  **Post-processing:** A land cover mask was used to convert the value of areas with high intensity development, barren, cultivated crops, and open water land covers to 0. Model outputs were clipped to the GGBN terrestrial boundary. |
